# Widefield Swept-Source Optical Coherence Tomography Angiography Assessment of Choroidal Changes in Vogt-Koyanagi-Harada Disease

**DOI:** 10.3389/fmed.2021.698644

**Published:** 2021-09-16

**Authors:** Yujing Qian, Jingyuan Yang, Anyi Liang, Chan Zhao, Fei Gao, Meifen Zhang

**Affiliations:** ^1^Department of Ophthalmology, Peking Union Medical College Hospital, Chinese Academy of Medical Sciences and Peking Union Medical College, Beijing, China; ^2^Key Laboratory of Ocular Fundus Diseases, Chinese Academy of Medical Sciences and Peking Union Medical College, Beijing, China

**Keywords:** Vogt-Koyanagi-Harada disease, widefield swept-source optical coherence tomography angiography, choroidal thickness, choroidal vascular index, choriocapillaris

## Abstract

**Purpose:** To investigate choroidal changes in patients with Vogt-Koyanagi-Harada disease (VKH) using widefield swept-source optical coherence tomography angiography (SS-OCTA).

**Methods:** In this cross-sectional study, 133 eyes of 69 patients with VKH (52 eyes of 28 active VKH patients and 81 eyes of 41 inactive VKH patients) and 104 eyes of 52 age and sex matched healthy volunteers were imaged using a widefield SS-OCTA instrument. On 12 mm × 12 mm OCTA scans, mean choroidal thickness (MCT), choroidal vascularity index (CVI), choriocapillaris (CC) flow area, and mean retinal thickness (MRT) were separately calculated in the fovea (diameter of 1 mm) and in concentric rings with different radii (1–3, 3–6, 6–9, and 9–12 mm).

**Results:** Eyes with active VKH showed significant increases in MCT, CVI, and MRT, and decreased CC flow area in all central and peripheral regions (0–1, 1–3, 3–6, 6–9, and 9–12 mm) than in the healthy eyes (*p* ≤ 0.01) and inactive VKH eyes (*p* < 0.05). Inactive VKH eyes only showed marked decrease in CC flow area in all regions compared with controls (*p* < 0.05). Flow voids were observed in 51 of 52 (98.1%) active VKH eyes and 50 of 81 (61.7%) inactive VKH eyes on 12 mm × 12 mm OCTA. The MCT of all regions was significantly correlated with age, disease duration, and disease activity, whereas CVI was associated with age and disease activity. The CC flow void was related to visual acuity in all regions (*p* < 0.05).

**Conclusion:** Widefield SS-OCTA enables a more comprehensive evaluation of chorioretinal changes in patients with VKH disease. Structural and vascular abnormalities are observed in both the central and peripheral choroid and are closely correlated with disease activity.

## Introduction

Vogt-Koyanagi-Harada disease (VKH), a multisystemic autoimmune disease, is one of the most common uveitis entities leading to blindness in China (1). It mostly affects young adults and is characterized by bilateral granulomatous panuveitis and extraocular manifestations, including meningismus, vitiligo, and poliosis (2). To date, autoimmunity against pigmented tissues in target organs throughout the body has been accepted as the main pathogenesis of VKH disease (3). In the eyes, the primary and most significant pathophysiology of VKH is diffuse stromal choroiditis, followed by inflammation spreading to the choriocapillaris, retina, vitreous, and anterior segment (4). If the disease is not recognized at an earlier stage and controlled effectively, frequent acute attacks and chronic intraocular inflammation could result in significant blood supply impairments and structural damage in the choroid and retina, both in the foveal and parafoveal regions (5). Consequently, this can cause permanent visual impairment and serious complications.

Fluorescein angiography and indocyanine green angiography have long been used as validating tools for diagnosing and evaluating VKH disease (6). Nevertheless, these two invasive modalities are time-consuming and non-quantitative, with potential dye-related risks, and are, therefore, unsuitable for regular follow-up. In recent years, a number of studies have revealed swept-source optical coherence tomography (SS-OCT) to be useful for monitoring intraocular inflammation both clinically and pathologically (7). Building on this rapid-developed platform, SS-OCT angiography (SS-OCTA) is proposed as an effective non-invasive tool. SS-OCTA can not only overcome the above mentioned limitations, but also offer other advantages, such as superiority in tissue penetration that would be particularly suitable for providing comprehensive information on full-thickness choroidal features (8, 9). Recently, this tool has been widely applied in clinical practice and scientific research to understand the pathophysiology of chorioretinal diseases, including central serous chorioretinopathy and polypoidal choroidal vasculopathy (10, 11). However, this novel technique is rarely used to delineate choroidal changes in VKH patients (12). Moreover, most of the previous OCTA observations in VKH patients have focused on limited areas, restricted mainly to macular scans of 3 × 3 mm and 6 × 6 mm. To the best of our knowledge, there is dearth of studies evaluating the vascular disturbances and structural alternations using such widefield imaging tool.

Therefore, the objective of this cross-sectional study was to investigate in detail the choroidal changes in VKH disease in a larger field of view. Toward this, a widefield SS-OCTA with a 12 mm × 12 mm scanning protocol was used to cover as much of the posterior pole as possible.

## Materials and Methods

### Study Participants

All patients presenting with VKH disease at the Department of Ophthalmology at Peking Union Medical College Hospital, China, between September 2019 and January 2021 were recruited. The diagnosis of VKH was made strictly following the criteria of the International Committee on Nomenclature by ophthalmologists with expertise in uveitis (13). Patients with the disease in the acute uveitic phase or chronic recurrent phase, manifesting as active anterior uveitis (cells and flare in the anterior chamber, mutton-fat keratic precipitates, iris nodules, etc.), vitritis, and/or posterior uveitis, were categorized as having active VKH. Patients in the convalescent phase without any evidence of disease activity signs were categorized as having inactive VKH. Uveitis terminology and anatomic classification were described by the Standardization of Uveitis Nomenclature (SUN) Working Group (14). Individuals with any systemic diseases (e.g., other autoimmune, infectious, and cardiovascular diseases), malignancy, ocular diseases except for VKH, ocular surgery history, large refractive error (>−6.0 D or >+4.0 D) were excluded. Healthy volunteers were matched with patients in terms of sex and age.

This study was approved by the Institutional Review Board of Peking Union Medical College Hospital and conducted according to the tenets of the Declaration of Helsinki. Informed written consents were obtained from all the participants enrolled in the study.

Sociodemographic and clinical characteristics were recorded. All participants underwent a detailed ophthalmic examination, including the logarithm of the minimum angle of resolution (logMAR) of best-corrected visual acuity (BCVA), intraocular pressure, slit-lamp biomicroscopy, and fundoscopy. Inflammatory manifestations in the anterior and posterior segments were evaluated after appropriate pupil dilatation.

### OCTA Image Analysis

OCTA images were acquired using a commercial SS-OCTA instrument (VG200, SVision Imaging, Ltd., Luoyang, China) equipped with a 1,050-nm-wavelength laser (10). OCTA was performed using a raster scan protocol of 512 (horizontal) × 512 (vertical) and 1,024 (horizontal) × 1,024 (vertical) B-scans, which covered an area of 3 mm × 3 mm and 12 mm × 12 mm centered on the fovea. The quality of the OCTA images was automatically graded by the built-in software from quality index Q1 (the worst) to Q10 (the best). Eyes with poor image quality (quality index <6) due to refractive media opacity or poor fixation were excluded.

For the quantitative analysis of 12 mm × 12 mm OCTA scans, choroidal and retinal parameters including mean choroidal thickness (MCT), choroidal vascularity index (CVI), choriocapillaris (CC) flow area, and mean retinal thickness (MRT) were separately calculated both in the central and peripheral regions, including the fovea (diameter of 1 mm) and the parafoveal (1–3 mm), perifoveal (3–6 mm), pararetinal (6–9 mm), and periretinal (9–12 mm) rings using built-in software version 1.28.11. Central foveal thickness (CFT) and subfoveal choroidal thickness (SFCT) were also automatically measured. The choroidal thickness was defined as the distance between the outer portion of the RPE line and the hyperreflective line behind the large choroidal vessel layers in response to the choroidal-scleral interface. This was taken as 1,000 μm in cases where the thickness was beyond the measurement limit and the inner surface of the sclera was not visible. CVI was defined as the ratio of the choroidal vascular luminal volume to the total choroidal volume, which reflects the volumetric choroidal vascular density (10, 15). The choriocapillaris was the region 10 μm above the Bruch membrane to 25 μm below it. The term choriocapillaris flow area was used for the area occupied by vessels in the choriocapillaris layer. Retinal thickness was considered as the vertical distance between the vitreoretinal interface and Bruch membrane. Layer segmentation and quantification analyses were performed automatically using the built-in software. Manual manipulation of segmentation was conducted to ensure accuracy, if necessary.

For qualitative analysis of the OCTA scans, the presence of flow voids on both 3 mm × 3 mm and 12 mm × 12 mm images was documented. Flow voids were defined as variable multifocal dark areas present throughout the scan, where the corresponding structural en face and cross-sectional OCTA images did not display any loss of signal transmission (16). Two trained doctors (YJQ and AYL; ophthalmologists with subspecialty training in the uveitis and retina) independently performed all the image evaluations in the study.

### Statistical Analysis

Statistical analysis was undertaken using SPSS (version 25.0; IBM Corp., Armonk, NY, USA). The Kolmogorov-Smirnov test was used for normality testing. Normal variables are presented as the mean and standard deviation (SD), and non-normal variables as the median and interquartile range (IQR). *t*-tests were performed to compare the means of normally distributed quantitative variables; otherwise, the Mann–Whitney *U*-test was used. Chi-square test was used to compare the qualitative data. Spearman correlation coefficients were employed to investigate the correlations between the OCTA metrics and clinical variables. The coefficient of variation was calculated as the standard deviation divided by the mean value. Differences were considered statistically significant at *p* < 0.05.

## Results

### Demographic and Clinical Features of Participants

A total of 133 eyes of 69 patients with VKH (23 men, 33.3%) and 104 eyes of 52 age- and sex-matched healthy volunteers (18 men, 34.6%) were enrolled in the study. Five VKH eyes with poor image quality were excluded. The sociodemographic and clinical features of all subjects are summarized in Table 1. The mean ages of the VKH patients and healthy controls were 37 (31–54) years and 38 (31–45) years, respectively. All participants included in this study were Han Chinese. Among 69 patients with VKH (133 eyes), 41 were inactive VKH patients (81 eyes), and 28 were active VKH patients (52 eyes). Patients with inactive/active VKH had worse LogMAR BCVA than healthy subjects (*p* < 0.001). Central foveal thickness and subfoveal choroidal thickness were significantly higher in eyes with active VKH than in the controls (*p* < 0.001). No significant differences in age, sex, and intraocular pressure were found between the inactive/active VKH patients and healthy controls (*p* > 0.05).

**Table 1:** Demographic and clinical features of healthy controls and patients with inactive/active VKH.

	**Healthy control (*n* = 52)**	**Inactive VKH (*n* = 41)**	***P* ^**a**^**	**Active VKH (*n* = 28)**	***P* ^**b**^**
Eyes	104	81		52	
Age (years), M (IQR)	38 (31–45)	37 (35–54)	0.239	34 (31–53)	0.960
Male, *n* (%)	18 (34.6)	15 (36.6)	0.844	9 (32.1)	0.823
LogMAR BCVA, M (IQR)	0 (0–0)	0.10 (0.00–0.30)	** <0.001**	0.30 (0.10–0.60)	** <0.001**
Intraocular pressure, M (IQR)	14.0 (13.0–16.0)	14.2 (13.1–18.4)	0.133	15.0 (12.9–16.0)	0.693
Duration of VKH (months), M (IQR)	–	12.0 (4.0–32.0)		3.5 (1.0–13.0)	
Bilateral involvement, *n* (%)	–	41 (100.0)		28 (100.0)	
Panuveitis, *n* (%)	–	41 (100.0)		28 (100.0)	
Central foveal thickness (μm), M (IQR)	218.0 (207.5–226.0)	213.0 (202.0–236.0)	0.476	247.0 (213.5–358.3)	** <0.001**
Subfoveal choroidal thickness (μm), M (IQR)	342.5 (279.3–439.5)	351.0 (265.0–438.0)	0.767	526.0 (380.0–721.8)	** <0.001**

*VKH, Vogt-Koyanagi-Harada disease; LogMAR, logarithm of the minimum angle of resolution; BCVA, best-corrected visual acuity.*

a *Comparisons between healthy controls and inactive VKH patients.*

b *Comparisons between healthy controls and active VKH patients.*

*Bold values: p < 0.05*.

### Quantitative Evaluation on 12 mm × 12 mm OCTA Scans

#### MCT

**Figure 1:**
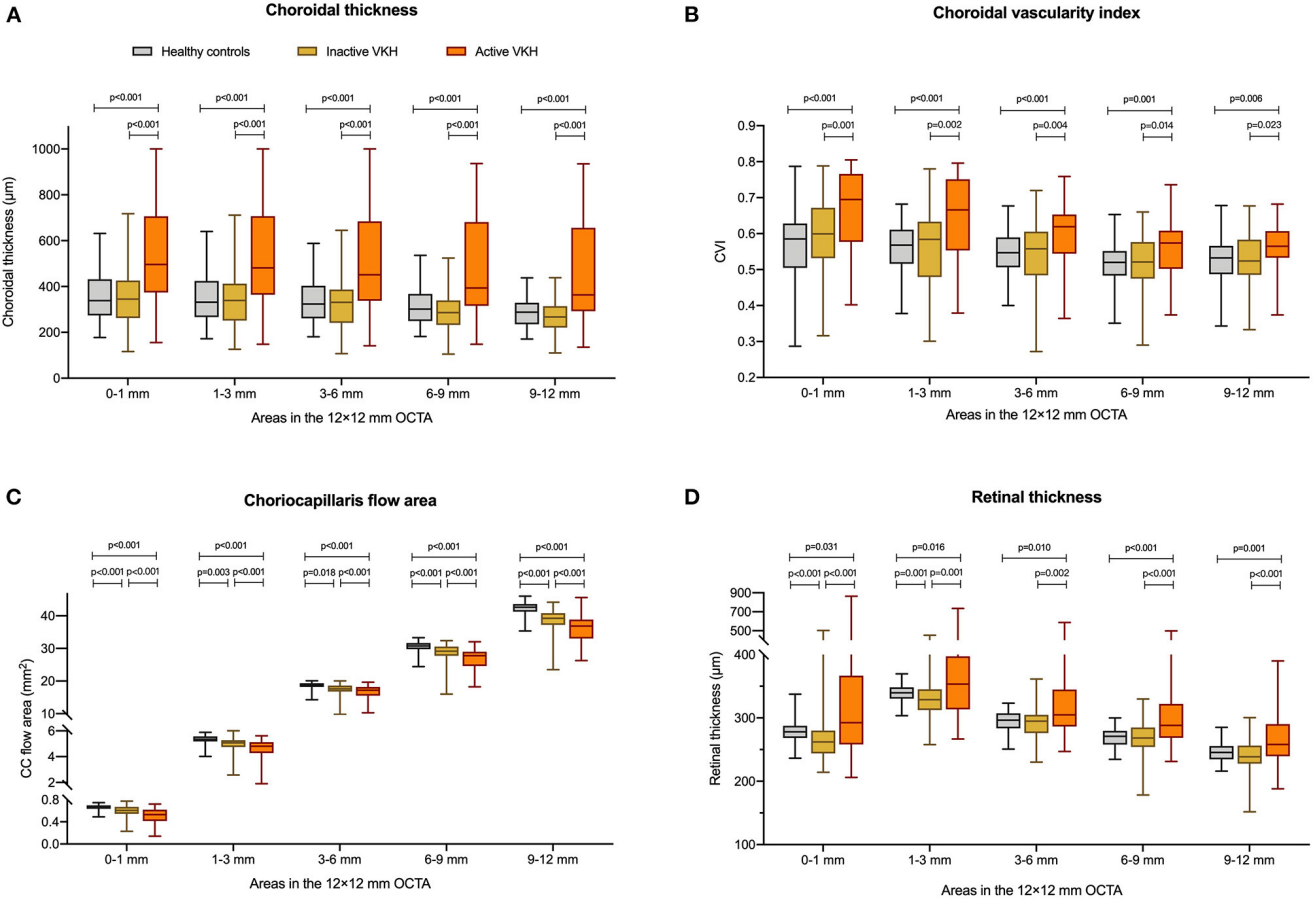
Comparisons of **(A)** choroidal thickness, **(B)** choroidal vascularity index (CVI), **(C)** choriocapillaris (CC) flow area, and **(D)** retinal thickness between healthy controls, inactive Vogt-Koyanagi-Harada (VKH), and active VKH patients in the central fovea (0–1 mm) and different radius of concentric rings (1–3, 3–6, 6–9, 9–12 mm) on the 12 mm × 12 mm swept-source optical coherence tomography angiography (SS-OCTA) scans. Data are shown in composite box plots. Whisker: minimum to maximum; box: interquartile range; lower and upper borders of box: the lower and upper quartiles (25th and 75th percentiles); line inside the box: median. The non-parametric Mann–Whitney *U*-test was used to compare the differences of parameters between groups.

**Figure 2:**
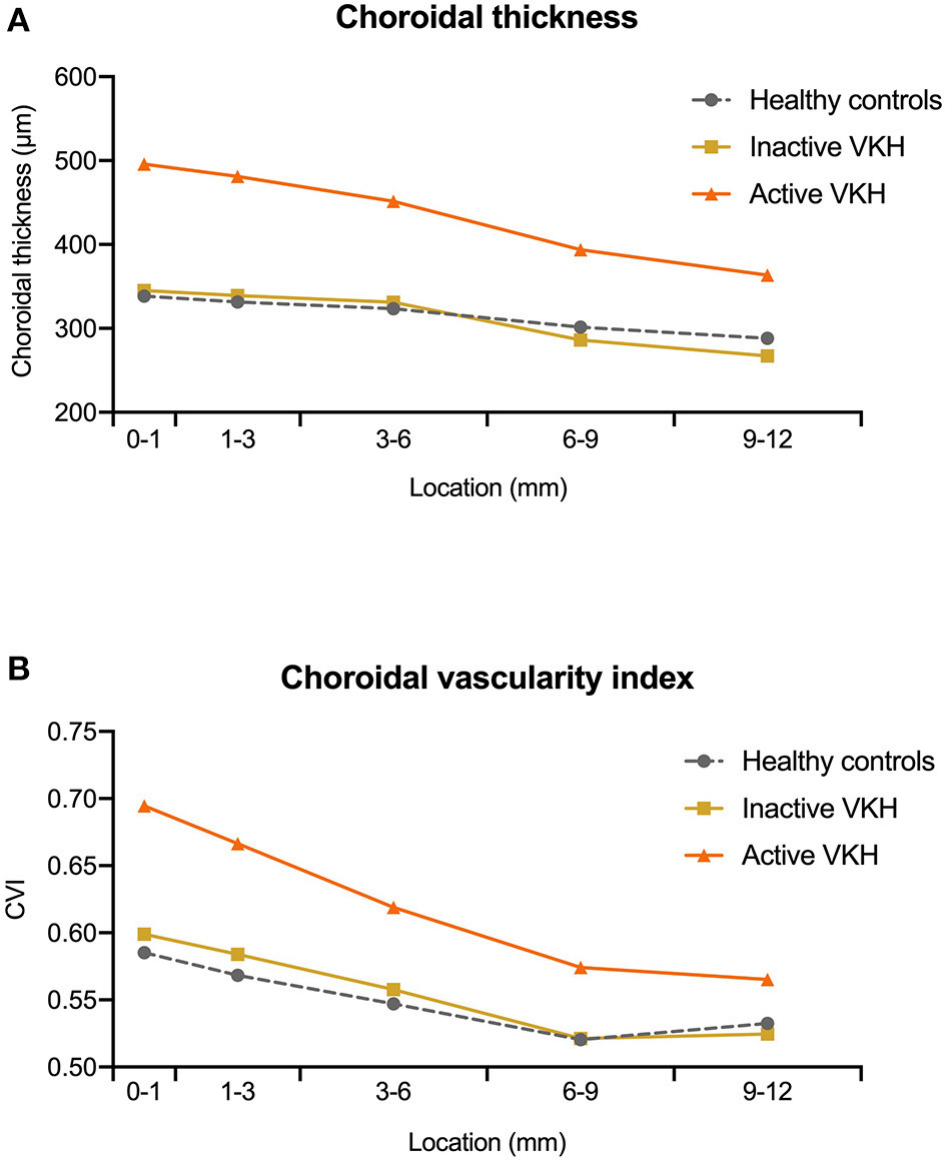
The median value of **(A)** choroidal thickness and **(B)** choroidal vascularity index (CVI) measured at different locations (0–1, 1–3, 3–6, 6–9, and 9–12 mm rings) within the 12 mm × 12 mm optical coherence tomography angiography (OCTA) scans in healthy controls, inactive Vogt-Koyanagi-Harada (VKH), and active VKH patients.

On the 12 mm × 12 mm OCTA scans, significant choroidal thickening was observed both in the central fovea (0–1 mm) and in different radii of concentric rings (1–3, 3–6, 6–9, and 9–12 mm) of active VKH eyes, as compared to the healthy controls (*p* < 0.001 for all) and inactive VKH eyes (*p* < 0.001 for all; Table 2 and Figure 1). Moreover, in the active inflammatory stage, the change in MCT was greater in the central region than in the peripheral region (Figure 2). In contrast, in all divided regions, there was no significant difference in the MCT between the eyes with inactive VKH and control eyes. The choroid of inactive VKH patients was slightly thickened in the posterior pole (0–1, 1–3, and 3–6 mm rings), whereas it was thinner in the mid-peripheral regions (6–9 and 9–12-mm rings) in comparison with the healthy controls (*p* > 0.05).

**Table 2:** Choroidal and retinal parameters of healthy, inactive VKH, and active VKH eyes on 12 mm × 12 mm OCTA.

	**Healthy control (104 eyes)**	**Inactive VKH (81 eyes)**	**Active VKH (52 eyes)**
**Mean choroidal thickness (μm), M (IQR)**			
0–1 mm	338.60 (273.67, 431.59)	345.16 (262.18, 426.01)	495.98 (373.87, 706.37)
1–3 mm	331.55 (266.82, 424.35)	339.24 (251.21, 412.55)	481.19 (364.24, 707.11)
3–6 mm	323.63 (261.21, 403.10)	331.06 (241.17, 387.03)	451.51 (337.66, 684.56)
6–9 mm	301.60 (249.55, 367.97)	286.21 (231.97, 339.53)	393.85 (315.40, 681.00)
9–12 mm	288.30 (235.07, 328.90)	267.25 (220.64, 314.58)	363.40 (292.16, 655.91)
CVI (%), M (IQR)^*^			
0–1 mm	58.52 (50.50, 62.80)	59.88 (53.16, 67.20)	69.45 (57.71, 76.62)
1–3 mm	56.83 (51.56, 61.12)	58.38 (47.95, 63.31)	67.63 (55.26, 75.11)
3–6 mm	54.71 (50.62, 58.95)	55.77 (48.45, 60.52)	61.89 (54.45, 65.32)
6–9 mm	52.03 (48.35, 55.17)	52.11 (47.44, 57.66)	57.41 (50.22, 60.84)
9–12 mm	53.24 (48.71, 56.58)	52.45 (48.48, 58.28)	56.51 (53.27, 60.71)
**CC flow area (mm** ^**2**^ **), M (IQR)**			
0–1 mm	0.67 (0.63, 0.70)	0.61 (0.54, 0.67)	0.53 (0.41, 0.62)
1–3 mm	5.34 (5.18, 5.57)	5.08 (4.75, 5.28)	4.81 (4.27, 5.13)
3–6 mm	18.82 (18.17, 19.33)	17.64 (16.77, 18.61)	17.21 (15.50, 18.19)
6–9 mm	30.74 (29.78, 31.68)	29.15 (27.74, 30.57)	27.79 (24.61, 29.00)
9–12 mm	42.61 (41.26, 43.63)	39.23 (37.20, 40.84)	36.85 (33.04, 38.82)
**Mean retinal thickness (μm), M (IQR)**			
0–1 mm	277.96 (268.23,287.51)	262.12 (243.84, 280.03)	292.40 (258.10, 366.70)
1–3 mm	339.55 (330.35, 348.34)	328.78 (312.46, 345.19)	353.28 (313.51, 397.32)
3–6 mm	296.44 (283.63, 307.25)	294.97 (276.10, 304.83)	304.75 (286.38, 344.71)
6–9 mm	270.91 (257.80, 279.49)	268.25 (254.13, 284.96)	288.15 (268.59, 322.13)
9–12 mm	245.54 (234.51, 255.75)	238.66 (227.67, 256.40)	258.13 (239.45, 290.30)

*VKH, Vogt-Koyanagi-Harada disease; OCTA, optical coherence tomography angiography; CVI, choroidal vascularity index; CC, choriocapillaris.*

* *Only 31 of 52 eyes in the active VKH group were able to delineate the choroid luminal area in the SS-OCTA images and compute for CVI*.

#### CVI

**Figure 3:**
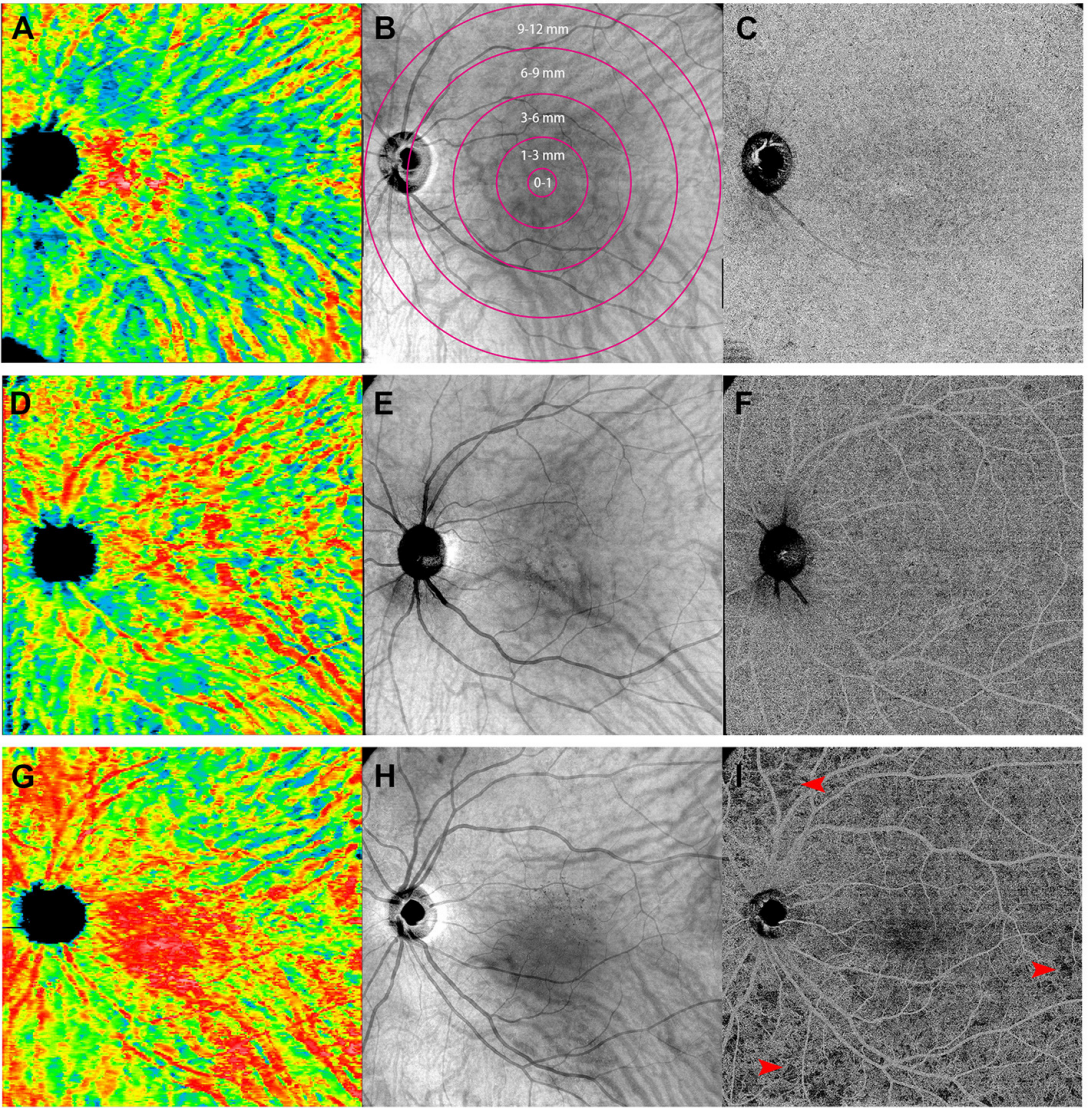
Representative of 12 mm × 12 mm three-dimensional choroidal vascularity index (CVI) maps (left: **A,D,G**), en face images at the level of choroid (middle: **B,E,H**), and the swept-source optical coherence tomography angiography (SS-OCTA) scans at the level of choriocapillaris (right: **C,F,I**) obtained from the left eye of a sex- and age-matched control (first row: **A,B,C**), an inactive Vogt-Koyanagi-Harada (VKH) patient (second row: **D,E,F**), and an active VKH patient (third row: **G,H,I**). The maps of three-dimensional CVI shows that as compared to the healthy eye **(A)**, choroidal vessels mildly dilated in the inactive VKH eye **(D)**, whereas significantly dilated in the active VKH eye **(G)**, especially in the central region. The OCTA image from a representative active VKH subject **(I)** shows multifocal flow void which were widespread in distribution and variably size at the level of choriocapillaris layer (red arrowheads).

Another useful index reflecting the status of the choroid, CVI was also markedly increased in all regions of the active VKH eyes, as compared to the healthy eyes (*p* < 0.01) and inactive VKH eyes (*p* < 0.05; Figure 3), similar to those in MCT. Furthermore, in eyes with active VKH, the increase in the CVI value was also greater in the central region than in the peripheral region. Consistent with the trend in MCT, CVI was slightly increased in most of the subfields of inactive VKH eyes, whereas it decreased in the periphery (9–12 mm ring), with no statistically significant difference (*p* > 0.05). Moreover, in 104 eyes of 52 healthy controls, the average CVI (52.90 ± 5.27%) of the entire 12 mm × 12 mm area showed a lower coefficient of variation (COV, 9.96%), indicating a greater stability as compared to the MCT (305.96 ± 70.30 μm, COV = 22.98%). Likewise, the COV of the average CVI was also significantly lower than that of the MCT (14.64 vs. 50.43%) in the eyes with VKH disease.

#### CC Flow Area

The active VKH group showed significant decrease in CC flow area in the fovea and all concentric rings, as compared to the control group (*p* < 0.001) and inactive VKH group (*p* < 0.001). The CC flow area was also significantly lower in the inactive VKH group than in the control group (*p* < 0.001).

#### MRT

The mean retinal thickness significantly increased in all regions of eyes with active VKH than in the healthy eyes (*p* < 0.05) and inactive VKH eyes (*p* < 0.01). In contrast, the MRT of every divided region was lower in the inactive VKH eyes than in the healthy controls, with statistically significant differences in the central foveal (0–1 mm circle) and parafoveal regions (1–3 mm ring) (*p* < 0.001 and *p* = 0.001, respectively).

### Qualitative Evaluation on OCTA Scans

At the choriocapillaris layer, of the 52 eyes with active VKH, a widespread distribution of flow voids of various sizes and shapes was observed in 51 eyes (98.1%) in the 3 mm × 3 mm OCTA images and in 48 eyes (92.3%) in the 12 mm × 12 mm OCTA images (*p* = 0.363). Of the 81 eyes with inactive VKH disease, 64 eyes (79.0%) showed flow voids in the 3 mm × 3 mm scans, whereas only 50 eyes (61.7%) showed a clear display of this feature in the 12 mm × 12 mm scans (*p* = 0.016).

### Correlation Analyses Between Clinical Parameters and OCTA Quantitative Measurements

Based on Spearman correlation analyses between the demographic and clinical indices of VKH patients and chorioretinal measurements of 12 mm × 12 mm OCTA scans, it was seen that age was negatively correlated with choroidal thickness (*p* ≤ 0.001) and CVI (*p* < 0.05) of all subfields (Table 3). Furthermore, age was also related to the CC flow area and retinal thickness in some of the divided regions (*p* < 0.05). In addition, it was found that disease duration was closely associated with choroidal thickness in all subfields, with an increasing correlation coefficient (*p* < 0.001), and with peripheral retinal thickness (*p* < 0.001). Furthermore, a negative correlation was found between LogMAR BCVA and CC flow area in all subfields of 12 mm × 12 mm images (*p* < 0.05). No other variables (such as sex, IOP) were found to be significantly associated with structural and vascular measurements on widefield OCTA scans (*p* > 0.05).

**Table 3:** Correlation analyses between clinical parameters and OCTA quantitative measurements in 69 patients with VKH disease.

	**Age**		**Disease duration**		**LogMAR BCVA**	
	**Rho**	***P***	**Rho**	***P***	**Rho**	***P***
**Choroidal thickness**						
0–1 mm	−0.277	**0.001**	−0.622	** <0.001**	0.134	0.125
1–3 mm	−0.288	**0.001**	−0.643	** <0.001**	0.133	0.126
3–6 mm	−0.313	** <0.001**	−0.655	** <0.001**	0.121	0.166
6–9 mm	−0.352	** <0.001**	−0.680	** <0.001**	0.123	0.160
9–12 mm	−0.376	** <0.001**	−0.702	** <0.001**	0.123	0.160
**CVI**						
0–1 mm	−0.200	**0.034**	0.037	0.696	−0.170	0.074
1–3 mm	−0.220	**0.020**	0.042	0.661	−0.178	0.061
3–6 mm	−0.239	**0.011**	0.007	0.943	−0.161	0.089
6–9 mm	−0.206	**0.029**	0.043	0.656	−0.150	0.115
9–12 mm	−0.264	**0.005**	−0.009	0.929	−0.150	0.114
**CC flow area**						
0–1 mm	−0.240	**0.005**	0.099	0.258	−0.174	**0.046**
1–3 mm	−0.228	**0.008**	0.129	0.137	−0.175	**0.044**
3–6 mm	−0.161	0.065	0.180	**0.038**	−0.175	**0.043**
6–9 mm	−0.124	0.154	0.199	**0.022**	−0.190	**0.028**
9–12 mm	−0.214	**0.013**	0.113	0.197	−0.219	**0.011**
**Retinal thickness**						
0–1 mm	0.065	0.455	0.078	0.372	0.114	0.191
1–3 mm	−0.003	0.970	0.058	0.504	0.069	0.431
3–6 mm	−0.098	0.261	−0.139	0.110	0.127	0.145
6–9 mm	−0.277	**0.001**	−0.407	** <0.001**	0.146	0.095
9–12 mm	−0.211	**0.015**	−0.431	** <0.001**	0.151	0.082

*VKH, Vogt-Koyanagi-Harada disease; OCTA, optical coherence tomography angiography; LogMAR, logarithm of the minimum angle of resolution; BCVA, best-corrected visual acuity; CVI, choroidal vascularity index; CC, choriocapillaris.*

*Bold values: p < 0.05*.

## Discussion

The choroid is recognized as the main target of autoimmunity in the pathogenesis of VKH disease (6). Diffuse inflammation in the choroid mostly affects the choroidal stroma and can secondarily damage the choriocapillaris. SS-OCTA, with a 1,050 nm tunable laser, has distinct advantages in the evaluation of choroidal involvement in VKH disease, as it can better visualize the choroidal and choriocapillaris layers with a greater span of the images (17, 18). However, before this study, we did not find any published cross-sectional studies that reported choroidal changes in VKH disease using widefield SS-OCTA.

Apart from the traditional choroidal vascular parameters, in the current study, we adopted the CVI (19) to quantitatively assess the status of choroidal vessels in patients with VKH. The three-dimensional SS-OCTA-derived CVI was separately calculated in different subfields of the 12 mm × 12 mm images. It was found that the CVI of active VKH patients was significantly higher in all regions, as compared to that of the inactive patients and healthy controls. These findings not only validated the results obtained from a small retrospective series (19), but also provided new evidence supporting the view that vascular dilatation and stasis of blood flow may occur in the choroid during active VKH inflammation (20). Furthermore, our results also revealed that the dilated vascularity in the active stage could decrease markedly in the inactive stage and reach a level similar to that of healthy individuals. Hence, we propose that CVI may represent a novel indicator for monitoring disease activity in VKH patients. In addition, as compared to the average MCT of the 12 mm × 12 mm area, the average CVI exhibited relatively lower variability in both healthy volunteers and patients with VKH. These observations were similar to those reported in previous studies (10, 15). Moreover, CVI could provide additional information regarding changes in vascular structure compared to conventional choroid thickness (21). Therefore, we agree with the view that CVI may be less easily affected by potential confounding factors (19). Hence, this could serve as a relatively more stable and novel measurement in evaluating choroid status.

In this study, the changes in MCT coincided well with CVI, be it with respect to comparisons between different subgroups of patients, or among comparisons between different subfields of the scanning area, indicating a satisfactory consistency of these two indices. Additionally, the MCT of all divided regions significantly correlated with age, disease duration, and disease activity of patients with VKH disease. Thus, we re-emphasize the irreplaceable role of MCT in monitoring VKH disease progression during follow-up.

Using a 12 mm × 12 mm scanning protocol to acquire the widefield image of the posterior pole, we uncovered the peripheral features of VKH eyes in the current study. Consistent results were obtained both in the central and peripheral regions of active VKH eyes, showing significant increases in the MCT, CVI, and MRT, and a decrease in CC flow area, as compared to the inactive VKH and control groups. In contrast, in eyes with inactive VKH, MCT and CVI were not significantly different from healthy eyes in all divided regions, and showed slight decreases in the peripheral region (Figure 2). Our results suggest that patients with VKH have significant choroidal thickening and luminal enlargement in the active stage, which gradually returned to near normal in the inactive stage, and could develop choroidal thinning and vessel shrinkage along with disease progression (22, 23). We noticed that the detection rate of flow void in inactive VKH eyes on the 12 mm × 12 mm scans was lower than that in the 3 mm × 3 mm scans. We believe that this may be partly related to the present technical limit of imaging resolution. Taken together, widefield OCTA is currently more suitable for screening and monitoring VKH disease. The application of this advanced technology facilitates a comprehensive evaluation of the structural and vascular changes both in the central and peripheral regions of the choroid, and broadens our understanding of the pathophysiology of VKH disease.

The present study has some limitations. First, the cross-sectional nature somewhat limited the dynamic assessment of the choroid changes as the disease develops and progresses. Further longitudinal prospective studies on widefield imaging as well as basic experimental studies are needed to validate the current results and provide novel information. Second, it would be of higher clinical relevance to conduct the study in a diverse study population using more types of OCTA devices. Third, to ensure accurate segmentation and consequently precise calculation of the measurements, we had to exclude some OCTA scans with ineligible imaging quality, which may have led to a selection bias.

Nevertheless, this is the first report that used widefield SS-OCTA to evaluate choroidal changes in a 12 mm × 12 mm field of view. The results revealed that disease activity correlated with changes in choroidal thickness (thickening/thinning) and vasculature (dilation/shrinkage), even in the peripheral choroid of VKH eyes.

In conclusion, this study provides comprehensive information and newer insights into the choroidal pathophysiology of VKH disease and highlights that widefield SS-OCTA can serve as a novel and valuable tool for the clinical assessment and scientific investigation of ocular diseases.

## Data Availability Statement

The raw data supporting the conclusions of this article will be made available by the authors, without undue reservation.

## Ethics Statement

The studies involving human participants were reviewed and approved by the institutional review board of the Peking Union Medical College Hospital. The patients/participants provided their written informed consent to participate in this study. Written informed consent was obtained from the individual(s) for the publication of any potentially identifiable images or data included in this article.

## Author Contributions

YQ, JY, and AL evaluated the images. YQ performed the data acquirement, analysis, interpretation, and wrote the manuscript. MZ, CZ, and FG recruited the patients. MZ critically reviewed the manuscript and provided valuable revisions to the manuscript. All authors made substantial contributions to the conception and design of this study, and read and approved the final manuscript.

## Funding

This work was supported by the National Natural Science Fund of China (Grant number 81770917 and 82070952).

## Conflict of Interest

The authors declare that the research was conducted in the absence of any commercial or financial relationships that could be construed as a potential conflict of interest.

## Publisher's Note

All claims expressed in this article are solely those of the authors and do not necessarily represent those of their affiliated organizations, or those of the publisher, the editors and the reviewers. Any product that may be evaluated in this article, or claim that may be made by its manufacturer, is not guaranteed or endorsed by the publisher.
